# Multipurpose Prevention Technologies: Opportunities and Challenges to Ensure Advancement of the Most Promising MPTs

**DOI:** 10.3389/frph.2021.704841

**Published:** 2021-09-06

**Authors:** Bethany Young Holt, Jim A. Turpin, Joseph Romano

**Affiliations:** ^1^Initiative for Multipurpose Prevention Technologies, CAMI Health, Public Health Institute, Sacramento, CA, United States; ^2^Howpin Consulting, Frederick, MD, United States; ^3^NWJ Group, LLC, Wayne, PA, United States

**Keywords:** multipurpose prevention technologies, hiv prevention, STI prevention, contraception, MPTs, sexual and reproductive health

## Introduction

The HIV/sexually transmitted infection (STI) syndemics and the unmet need for modern contraceptive methods continue to pose significant health risks for women worldwide (1). Women are at high risk of HIV in many regions of the world, particularly young women and girls in Sub-Saharan Africa where 71% of all new infections are among adolescents (2–4). The risk of HIV acquisition and transmission through mother-to-child transmission (MTCT) and among pregnant and breastfeeding women are significant contributors to the HIV epidemic (5). Rates of STIs, such as gonorrhea, chlamydia, syphilis, and herpes are rising and compounding the risk of HIV acquisition (6, 7). At the same time, an estimated 218 million women in lower- and middle-income countries (LMICs) have an unmet need for contraception (8), and 817 women die each day from preventable causes related to pregnancy or child-birth (7). As awareness of the need to address these interlinked risks has increased, the need for new technologies that combine protection against unintended pregnancy, HIV and other STIs is a growing research priority (9). Addressing these interlinked risks also aligns with the goals of the WHO-led initiative for the elimination of maternal-to-child transmission (EMTCT) of HIV and syphilis as a public health priority (5).

Multipurpose Prevention Technologies (MPTs) are products that simultaneously prevent HIV, other STIs, and/or unintended pregnancy. They have power to revolutionize women's health by providing prevention for multiple indications (10). Additionally, MPTs that combine HIV prevention and contraception may improve uptake of and adherence to HIV pre-exposure prophylaxis (PrEP) by offering streamlined product delivery and eliminating the need for multiple, separate clinic visits to address family planning and other sexual and reproductive health (SRH) needs (11). Given the urgent need to reduce HIV infection in pregnant and breastfeeding women, MPTs that allow for contraception and prevent HIV and other STIs may benefit women who wish to conceive as well as pregnancy and breast feeding women who are not using contraceptives. A study among pregnant women of the dapivirine ring and the Truvada oral tablet aims to assess the safety, adherence and acceptability of these HIV prevention approaches when used during pregnancy which can inform MPT development (12). Yet, even with the potential of MPTs to transform the lives of women everywhere, especially those in LMICs, MPT development is scientifically and logistically complex. The resources critical for transitioning promising pre-clinical product candidates and formulations into clinical evaluation remain limited despite intensified collaborations and investments between government and private sectors.

## Opportunities

Male and female condoms, when used properly through their multi-spectrum ability to block infection and transmission of HIV and other STIs and prevent pregnancy, are the only currently approved MPT products. The United Nations (UN) Department of Economic and Social Affairs recently reported a worldwide increase in condom use from 1994 to 2015 (13). The global condom market was valued at $6.76 billion in 2017 and is estimated to increase to $11.1 billion by 2023 (14). Yet, for many people condoms are not feasible for a variety of reasons. These include coital dependency, unequal power balance between male and female partners and other challenges that may arise in negotiating condom use in intimate relationships, and sexual preferences that play into condom disuse. Despite these many challenges, alternative contraceptive use and acceptability have markedly increased since the creation of various modern methods [such as the pill and the intrauterine device (IUD)] (13). However, the most commonly used contraceptive methods, namely sterilization, the pill, injectables and IUDs, do not protect against STIs. Thus, condoms are only a precursor to other successful MPTs, in which various drug delivery platforms (e.g., pills, injectables, vaginal rings, and subdermal implants etc) can be leveraged as more user-friendly options.

**Figure 1:**
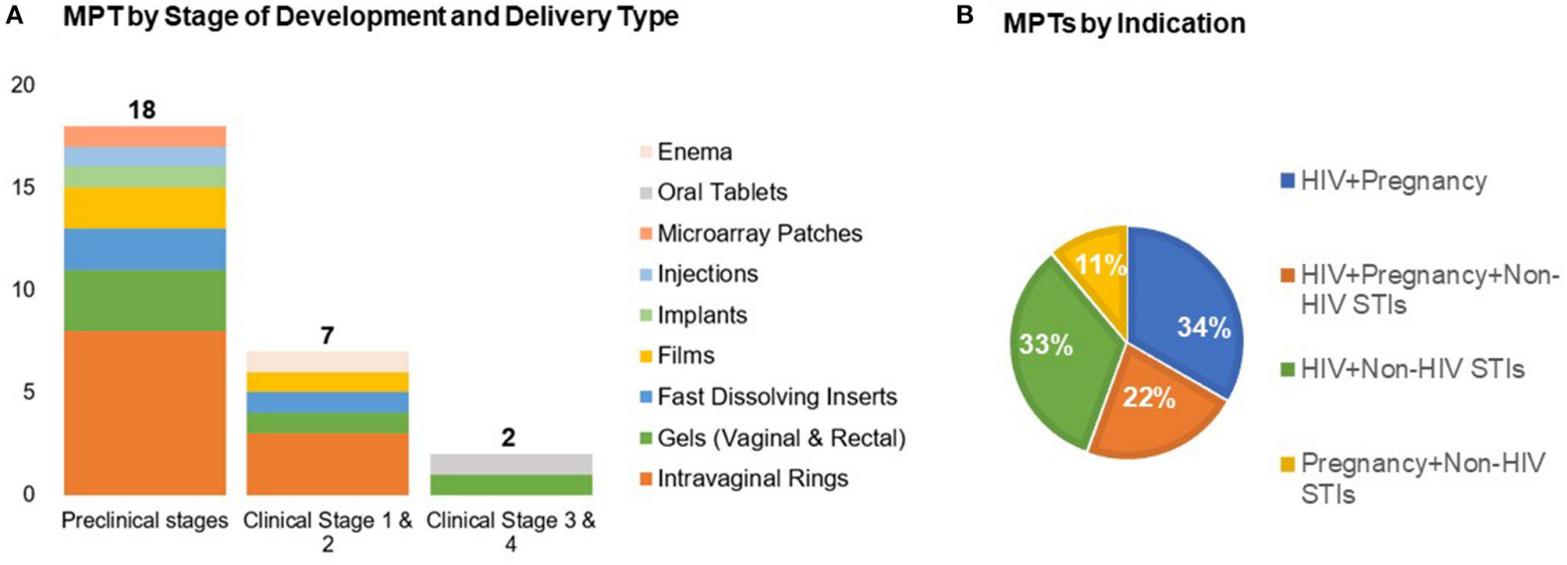
Recent advances in MPTs development by **(A)** stage of development across delivery types and **(B)** indication (*n* = 27). Adapted from the Initiative for MPTs (IMPT) pipeline database (15).

Over the past decade there has been a growing array of new MPT candidates proposed, with over two dozen in active development. These include intravaginal rings, vaginal and rectal gels, vaginal inserts and films, systemic delivery implants, subdermal microarray patches, and oral tablets containing contraceptives, anti-HIV and/or other STI prevention drugs (15) (Figure 1). The majority of MPT candidates are in early pre-clinical stages of development by small biotechnology companies and academic labs. These efforts are largely funded by the United States government, primarily the United States Agency for International Development (USAID) and the National Institutes of Health (NIH).

Multiple end-user preference studies are lending support to the potential reproductive health impact of MPTs, as well as informing on product design strategies. For example, an end-user study conducted in three sub-Saharan Africa countries indicates specific end-user preferences identifying preferred MPT product dosage forms (16). The TRIO Study explored user preferences after use of placebo formulations of oral tablets, intravaginal rings and injectables (17). Further insight into the potential success of long acting MPTs can also be inferred from a study of preference for long-acting injectable PrEP conducted among African and US women (18). In the US, several studies have specifically gauged women's preferences for MPTs (19, 20), including a cross-sectional national survey that assessed women's preferences for MPTs in the form of injectables, vaginal gels, intravaginal rings and diaphragms. Results from these studies indicate a high level of end-user preference for female controlled, discreet, long-acting products, such as injectables. These finding are harmonious with decades of research on contraceptive methods demonstrating use is greater when more method options are available (21). Providing women with an array of MPT method options must then be a goal of any long term MPT development efforts and is essential in creating an MPT product portfolio that realizes the reproductive health impact of MPTs.

Although a start has been made in creating a new generation of MPT products, significant funding is required to bring these MPTS to market. This includes funds to support pre-clinical research for translation to clinical testing, support of clinical testing and small- and large-scale manufacturing. Importantly, this also includes investments of funds, time and expertise to address the complex regulatory requirements that will enable multi-indication MTPs to advance to regulatory approval. Because of this complexity, public and private partnerships between academic researchers, small companies, big pharmaceuticals, the USG, and other supporting groups are needed to foster an end- to-end approach while promoting the advancement of economically viable end-user friendly MPT products. Although resources remain limited, support for MPTs is growing within the U.S. Health and Human Services (22) as well as among European funders and life science investors. Many funding agencies that support MPTs are working to leverage and optimize limited resources to address priorities and gaps to advance the most promising candidates; such strategic collaborations are essential.

The current MPT field is building upon lessons learned from decades of research in contraception, HIV topical microbicide development, systemic HIV prevention products and prevention of non-HIV STIs. A number of single indication contraceptive products are available to women (23–25) and new innovations are also underway for male contraception (25, 26). Single indication HIV-only prevention products are also in development. Importantly, after multiple clinical trials demonstrating efficacy, the dapivirine intravaginal ring for HIV prevention is in the process of gaining regulatory approval in individual countries (27–30). Furthermore, long-acting injectable cabotegravir (CAB-LA) has completed phase 3 trials in men who have sex with men, transgender women, and cisgender women, showing strong efficacy and safety results (31, 32). Likewise, a growing number of HIV prevention combination products are in development that include two oral PrEP compounds approved for use: Truvada® for use by men and women containing emtricitabine and tenofovir disoproxil fumarate (33, 34), and Descovy®, containing emtricitabine and tenofovir alafenamide, which currently is only approved for men who have sex with men (MSM) and transgender women (35). New approaches for prevention of non-HIV STIs include those which address growing concerns around the development of antibiotic resistant STI prevention (36, 37). These include agents which stimulate host immune responses as well as non-immune approaches, including vaginal barrier methods, vaginal biofilm inhibitors, and microbiome modulators (38–42). As with contraceptives, all of these single indication anti-infective products and combination HIV prevention products can be critical components of a future MPT strategy since they can serve as the building blocks for multi-indication MPTs.

## Challenges

The increasing number of technology options and new drugs entering the prevention pipeline will require proper investment to confirm that adequate resources are available to support potential high impact products through licensure and access needs. Challenges include obstacles to manufacturing (e.g., mixing of hormone and non-hormone drug substances/products), clinical trials (e.g., design of Phase 3 multi-indication trials) and other regulatory challenges (e.g., requirements for licensure of a combination dual indication long-acting product in a potentially novel drug-delivery system). Future trials will need to move beyond placebo-controlled designs toward superiority and non-inferiority trials that compare any new candidate against existing effective treatment options. As more single indication prevention products become available, the less likely it will be that regulatory bodies will approve efficacy studies that are placebo controlled. This approach will require larger more expensive trials with more challenging logistics, particularly if products like the CAB-LA HIV prevention injectable or EFDA implants become the standard of care (SOC) for HIV prevention (at least for LA systemic products) (42, 43). Further, although concurrent development of promising MPT candidates can accelerate progress for the field, available limited resources should be invested in a portfolio of diverse promising approaches for indication, mechanism of action and dosage form, and avoid developing nearly identical MPT products without strong justification for such an investment.

Promoting the development of a product pipeline that combines and optimizes the expertise currently associated with single indication products to create the desired multi-indication MPTs is key. This will require integration of the preclinical, clinical manufacturing and regulatory expertise associated with STI and contraceptive product development into a focused platform capable of supporting the development and licensure of multi-indication MPTs. This effort will require not only clarifying the complex manufacturing and regulatory challenges associated with combining multiple drugs and excipients, that may have incompatible biophysical, rheological and biochemical properties, but also the creation of multidisciplinary public/private partnerships to fund and guide this effort. Central, too, for the success of MPTs is the technical guidance required to evaluate and advance promising preclinical products into clinical formulations that can be advanced to human testing and ultimately licensure, particularly for groups with little experience in these areas (44, 45).

## Discussion

MPTs present significant reproductive health and general opportunities for addressing multiple indications in at-risk populations, particularly adolescent girls and young women in regions of the world where risk of HIV, other STIs and unintended pregnancies remains high. Given the current limited resources for expansion of MPT product development, ongoing strategic thinking and action is needed to optimize use of technical capacities, enhance collaborative approaches, identify resources to help fill gaps, and add rigor to the development process with the aim of advancing the most promising products.

## Author Contributions

All authors listed have made a substantial, direct and intellectual contribution to the work, and approved it for publication.

## Author Disclaimer

The contents of this manuscript are the sole responsibility of the authors and do not necessarily reflect the views of USAID, NICHD or the United States Government.

## Conflict of Interest

BYH was employed by the Public Health Institute. JT was employed by the company Howpin Consulting. JR was employed by the company NWJ Group, LLC.

## Publisher's Note

All claims expressed in this article are solely those of the authors and do not necessarily represent those of their affiliated organizations, or those of the publisher, the editors and the reviewers. Any product that may be evaluated in this article, or claim that may be made by its manufacturer, is not guaranteed or endorsed by the publisher.
